# Genomic Basis of Transcriptome Dynamics in Rice under Field Conditions

**DOI:** 10.1093/pcp/pcab088

**Published:** 2021-06-16

**Authors:** Makoto Kashima, Ryota L Sakamoto, Hiroki Saito, Satoshi Ohkubo, Ayumi Tezuka, Ayumi Deguchi, Yoichi Hashida, Yuko Kurita, Koji Iwayama, Shunsuke Adachi, Atsushi J Nagano

**Affiliations:** Research Institute for Food and Agriculture, Ryukoku University, Yokotani 1-5, Seta Oe-cho, Otsu, Shiga 520-2194, Japan; Seibi Senior High School, Shohojicho 33, Gifu 500-8741, Japan; Graduate School of Agriculture, Kyoto University, Kitashirakawa-oiwake, Sakyo-ku, Kyoto 606-8317, Japan; Tropical Agriculture Research Front, Japan International Research Center for Agricultural Sciences, Maezato 1091-1, Ishigaki, Okinawa 907-0002, Japan; Graduate School of Agriculture, Kyoto University, Kitashirakawa-oiwake, Sakyo-ku, Kyoto 606-8317, Japan; Institute of Global Innovation Research, Tokyo University of Agriculture and Technology, Saiwaicho 3-5-8, Fuchu, Tokyo 183-8509, Japan; Research Institute for Food and Agriculture, Ryukoku University, Yokotani 1-5, Seta Oe-cho, Otsu, Shiga 520-2194, Japan; Research Institute for Food and Agriculture, Ryukoku University, Yokotani 1-5, Seta Oe-cho, Otsu, Shiga 520-2194, Japan; Research Institute for Food and Agriculture, Ryukoku University, Yokotani 1-5, Seta Oe-cho, Otsu, Shiga 520-2194, Japan; Research Institute for Food and Agriculture, Ryukoku University, Yokotani 1-5, Seta Oe-cho, Otsu, Shiga 520-2194, Japan; Faculty of Data Science, Shiga University, Bamba 1-1-1, Hikone, Shiga 522-0069, Japan; Institute of Global Innovation Research, Tokyo University of Agriculture and Technology, Saiwaicho 3-5-8, Fuchu, Tokyo 183-8509, Japan; Faculty of Agriculture, Ryukoku University, Yokotani 1-5, Seta Oe-cho, Otsu, Shiga 520-2194, Japan; Institute for Advanced Biosciences, Keio University, 403-1 Nipponkoku, Daihouji, Tsuruoka, Yamagata 997-0017, Japan

**Keywords:** Environmental response, eQT, Oryza sativa, RNA-Seq, Statistical modeling

## Abstract

How genetic variations affect gene expression dynamics of field-grown plants remains unclear. Expression quantitative trait loci (eQTL) analysis is frequently used to find genomic regions underlying gene expression polymorphisms. This approach requires transcriptome data for the complete set of the QTL mapping population under the given conditions. Therefore, only a limited range of environmental conditions is covered by a conventional eQTL analysis. We sampled sparse time series of field-grown rice from chromosome segment substitution lines (CSSLs) and conducted RNA sequencing (RNA-Seq). Then, by using statistical analysis integrating meteorological data and the RNA-Seq data, we identified 1,675 eQTLs leading to polymorphisms in expression dynamics under field conditions. A genomic region on chromosome 11 influences the expression of several defense-related genes in a time-of-day- and scaled-age-dependent manner. This includes the eQTLs that possibly influence the time-of-day- and scaled-age-dependent differences in the innate immunity between Koshihikari and Takanari. Based on the eQTL and meteorological data, we successfully predicted gene expression under environments different from training environments and in rice cultivars with more complex genotypes than the CSSLs. Our novel approach of eQTL identification facilitated the understanding of the genetic architecture of expression dynamics under field conditions, which is difficult to assess by conventional eQTL studies. The prediction of expression based on eQTLs and environmental information could contribute to the understanding of plant traits under diverse field conditions.

## Introduction

Organisms respond to fluctuations in field environments variably, depending on their genetic backgrounds, developmental stages and physiological status. This variability can cause missing heritability in crop breeding and low accuracy in medicine (Eichler et al. 2010, Brachi et al. 2011, Wang et al. 2017). Environmental stimuli can induce transcriptional responses directly and/or indirectly (Kato et al. 2019, Ohnishi et al. 2019, Savchenko et al. 2019, Abdelrahman et al. 2020, Nakayama et al. 2020, Xie and Chen 2020, Mao et al. 2020). Measuring transcriptome dynamics is a comprehensive method for assessing environmental responses and their alteration due to genetic, developmental and physiological factors (Han et al. 2020, Palit et al. 2020, Ye et al. 2020). The expression quantitative trait loci (eQTLs) approach is frequently used to assess the association between genetic variation and gene expression polymorphism (Jansen and Nap 2001, Wang et al. 2010, 2014, Horiuchi et al. 2015, Kuroha et al. 2017). Because this approach requires transcriptome data for the complete set of a QTL mapping population under the given conditions (Jansen and Nap 2001, Wang et al. 2010, 2014, Horiuchi et al. 2015, Kuroha et al. 2017), only a limited range of environmental conditions are covered. Although dense time-series eQTL analysis can extend the genetic and environmental cover range of eQTL, such study requires numerous samples and will be unwieldy to perform. Conversely, statistical models based on meteorological data, circadian clock, and age-in-days have succeeded in describing transcriptome dynamics in the field (Nagano et al. 2012, Matsuzaki et al. 2015, Iwayama et al. 2017), although such models can only predict the transcriptome of one or a few genotypes used as training data (Nagano et al. 2012, Matsuzaki et al. 2015, Iwayama et al. 2017).

Rice (*Oryza sativa*), a major crop in the world, has a sequenced genome (Kawahara et al. 2013, Sakai et al. 2018) and abundant genetic resources are available (Huang et al. 2012). ‘Takanari’ is a high-yield, indica cultivar with some indica-specific features, such as strong lodging resistance (Ookawa et al. 2016) and high photosynthetic rate (Takai et al. 2014). In addition to these attractive features, useful genetic resources such as CSSLs and backcross-inbred lines (BILs) between ‘Takanari’ and ‘Koshihikari’ (a leading cultivar in Japan) are available (Adachi et al. 2014, Takai et al. 2014). These resources are useful for QTL mapping of these beneficial traits.

In this study, to reveal loci relating to varietal differences in gene expression, we integrated conventional eQTL analysis and a statistical modeling approach based on meteorological data and RNA-Seq data. This novel approach identified eQTL that can elucidate genetic variation and transcriptome dynamics under fluctuating field conditions.

## Results

**Fig. 1:**
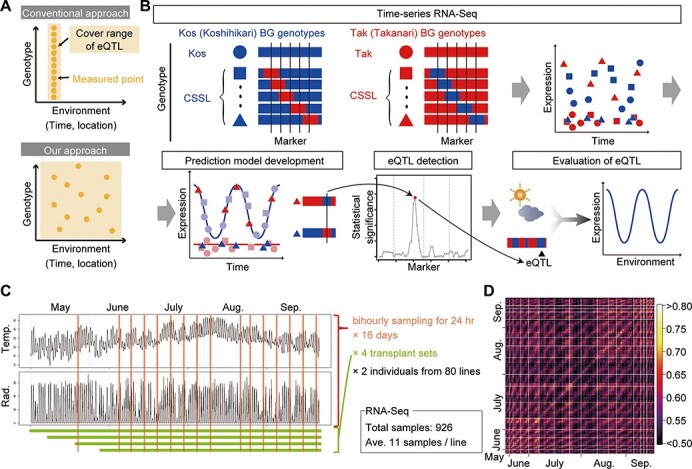
Concept and workflow of eQTLs detected in this study. (A) Conceptual differences in the cover range of eQTLs identified with the conventional and our novel approach. (B) Workflow of eQTL detection and its evaluation using CSSLs. (C) Summary of the sampling design for eQTL detection. The left panels show plots of meteorological data [air temperature (Temp., °C) and global solar radiation (Rad., kJ m^−2^ min^−1^)] in Takatsuki from May to September in 2015. Vertical red lines represent the sampling time points. (D) Pearson’s correlations among the 854 samples used for developing the prediction model based on expression data of 23,294 expressed genes. White lines indicate the border of each bihourly sampling set.

We identified eQTLs that determine polymorphisms in gene expression dynamics under fluctuating field conditions. To extend the environmental cover range of eQTL, we leveraged statistical modeling based on meteorological data and sparse time-series RNA-Seq of variable genotypes (**Fig. 1A** and **Supplemental Fig. S1**). Our approach consists of four steps: time-series RNA sequencing (RNA-Seq), prediction model development, eQTL detection, and evaluation of eQTL (See Methods, **Fig. 1B**, and **Supplementary Fig. S1**). We used the rice (*O. sativa* L.) cultivars ‘Koshihikari’ and ‘Takanari’, which would exhibit substantial polymorphisms in expression dynamics. Seventy-eight reciprocal CSSLs and two BILs developed from the parental lines were also used (Adachi et al. 2013, 2019, Takai et al. 2014) (**Fig. 1B** and **Supplementary Fig. S2A**). To examine the effect of plant age (days after seeding) in our model, we prepared four different sets of rice plants transplanted at 2-week intervals. Sixteen sets (days) of bihourly sampling for 24 h were conducted from May to September 2015 (cropping season in Japan). At each sampling time, the youngest fully expanded leaves were collected from two different genotypes in each transplant set, while withered plants (gray points in **Supplementary Fig. S2B**) were excluded from the sampling (**Fig. 1C**, **Supplementary Fig. S2B**, and **Table S2**). Nine hundred and twenty-six individual leaves were used in RNA-Seq analyses to obtain transcriptome data. After filtering samples and genes (**Supplementary Fig. S3A, B**) and confirming genotypes (**Supplementary Figs. S3C** and **4**, and supplementary information), 23,924 expressed genes from 845 samples were used. Correlation analysis of the transcriptome data showed an obvious transcriptome-wide diurnal variation in the genes expressed (**Fig. 1D**).

**Fig. 2:**
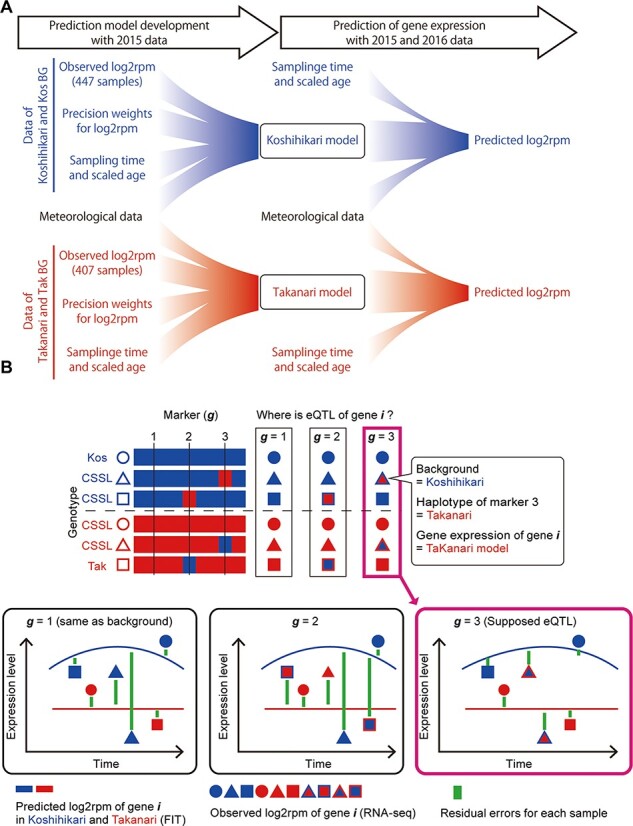
eQTL detection in this study. (A) Predictive models of gene expression for ‘Koshihikari’ and ‘Takanari’ were developed with ‘FIT’ based on RNA-Seq data (observed log_2_rpm), the corresponding precision weights, meteorological data and scaled age. Then, based on the models with input of meteorological data and sample attributes, predicted log_2_rpm of ‘Koshihikari’ and ‘Takanari’ can be obtained. (B) The association between genetic variations and gene expression polymorphisms was evaluated by calculating the sum of residual errors in gene expression prediction. It was assumed that the type of each gene expression dynamics is determined by SSR markers. The color of the enclosing lines of the circles, triangles and quadrangles indicate the BGs of each line. The fill color of the circles, triangles and quadrangles indicate which ‘Koshihikari’ or ‘Takanari’ model is used to predict gene expression. In the example, eQTL affecting gene *i* exist around SSR marker 3. In this case, the sum of residual errors on the assumption that eQTL is SSR marker 3 is smaller than the other residual errors.

**Fig. 3:**
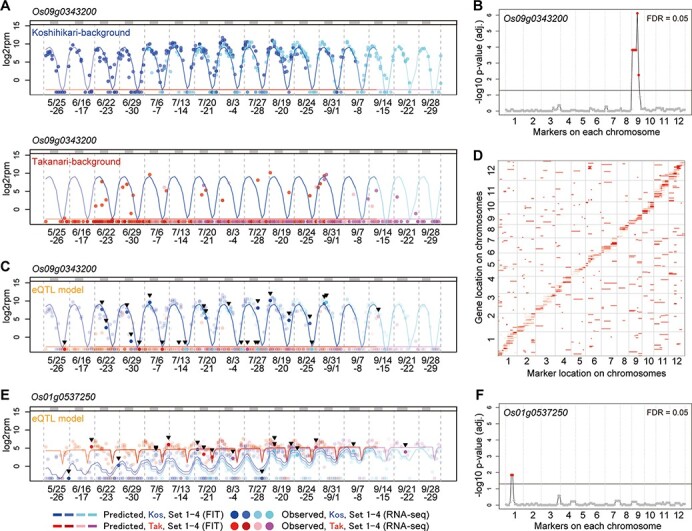
eQTL detection revealed *cis*- and *trans*-eQTLs that were involved in environmental responses. A, C, E, Observed and predicted expressions (log_2_rpm) of *Os09g0343200* (A, C) and *Os01g0537250* (E) in Takatsuki in 2015. The predicted expressions were calculated using ‘FIT’ based on scaled age and environmental information (time, air temperature and global solar radiation). Blue and red/pink lines indicate predicted expression levels in ‘Koshihikari’ and ‘Takanari’ in transplant sets 1, 2, 3 and 4, respectively. Blue and red/pink points indicate the expression level obtained by RNA-Seq for samples in transplant sets 1, 2, 3 and 4 of individuals with ‘Koshihikari’ BG and ‘Takanari’ BG in (a) or ‘Koshihikari’- and ‘Takanari’-type eQTL in (C, E) respectively. In (C, E) strong colored points emphasized by the arrowheads indicate samples harboring eQTLs different from their BGs, and light-colored points indicate the samples harboring eQTLs identical to their BGs. The upper gray bar indicates dark periods (global solar radiation < 0.3 kJ m^−2^ min^−1^). (B, F) eQTLs regulating *Os09g0343200* (B) and *Os01g0537250* (F). (D) Position of the 1,675 eQTLs are shown as red bars (false discovery rate = 0.05). X-axis and Y-axis represent the positions of markers with eQTLs and the positions of genes influenced by eQTLs, respectively.

We then developed two prediction models describing transcriptome dynamics under fluctuating field environments in ‘Koshihikari’ and ‘Takanari’ (**Fig. 2A**). In this step, we used the statistical modeling tool ‘FIT’ (Iwayama et al. 2017) for predicting transcriptome dynamics under field conditions. As most of the genome in the CSSLs was not substituted (**Supplementary Fig S2A**), the expression dynamics of most genes was expected to be identical to that of the background parent. Thus, the RNA-Seq data of ‘Koshihikari’- and ‘Takanari’-background samples were used to develop the prediction model for ‘Koshihikari’ and ‘Takanari’ lines, respectively (**Fig. 2A**). Circadian clock and meteorological data (air temperature and global solar radiation) (**Fig. 2A**) were considered in model development and ‘scaled age’ was used to adjust differences in heading date among rice genotypes (See Methods, **Fig. 2A** and **Supplementary Figs. S5** and **6**). We found polymorphisms in the predicted expression dynamics of 3,696 genes (15.4% of the expressed genes; **Supplementary Fig. S3E** and **Table S3**). For instance, the ‘Koshihikari’ model for *Os09g0343200* showed obvious diurnal oscillations in expression, whereas the ‘Takanari’ model showed a constant low level of expression (**Fig. 3A**). Notably, in some ‘Takanari’-background CSSLs, SL1329 and SL1330, the expression of *Os09g0343200* resembled that of the ‘Koshihikari’ model rather than that of the ‘Takanari’ model (**Fig. 3A**), suggesting that genetic substitution in the chromosome 9 affected *Os09g0343200* expression.

The CSSLs had been previously genotyped using 141 simple sequence repeat (SSR) markers (**Supplementary Fig. S2A**) (Takai et al. 2014). As so, at the eQTL detection step, we searched for the SSR markers explaining the expression dynamics polymorphisms. We evaluated the decrease in the residual error of each gene assuming eQTL around each SSR marker (See Methods and **Fig. 2B**). For *Os09g0343200*, the sum of residual errors significantly decreased only by assuming eQTL on chromosome 9 (**Fig. 2B** and **Supplementary Fig. S7A**), indicating that it was a *cis*-eQTL. The prediction models chosen for each sample based on eQTL genotypes explained the expression dynamics of *Os09g0343200* better than their background genotypes (**Fig. 3C**). SL1329 and SL1330 commonly harbor a Koshihikari-type chromosomal segment, distinguishable by the SSR marker RM3907 at 10.4 M bp on chromosome 9 (**Supplementary Table S1**). This suggests that polymorphism around RM3907, in Koshihikari and Takanari, would affect *Os09g0343200*, which is located at 10.6 Mb on chromosome 9. Overall, eQTLs were identified for 1,675 genes (45.3% of genes with expression dynamics polymorphism between ‘Koshihikari’ and ‘Takanari’, false discovery rate = 0.05; **Supplementary Fig. S7E**), including 222 genes affected by *trans*-eQTL (**Fig. 3D**). Forty-three genes were affected by multiple eQTLs. For 33 of these genes, the sum of residual errors based on all eQTLs was lower than that of the most significant eQTL alone (**Supplementary Fig. S8**). A *cis*-eQTL was identified for *Os02g0280700* (*HIS1*), which could contribute to the differences in resistance against a popular herbicide, benzobicyclon, between Koshihikari and Takanari (Akasaka et al. 2011, Maeda et al. 2019). Detailed results for individual genes can be found in our database (https://ps.agr.ryukoku.ac.jp/osa_eQTL). Among the 3,696 genes with expression polymorphisms, we identified 1,348 genes whose expression fluctuated depending on time of day either in ‘Koshihikari’ or ‘Takanari’ (**Supplementary Fig. S10**). Genes involved in the oxidation–reduction process were significantly enriched in the 1,348 genes (Gene Ontology (GO): 0055114, adjusted *P*-value = 6.83E-04, **Supplementary Table S4**). Also, 1,139 genes were identified whose expression fluctuated depending on scaled age either in ‘Koshihikari’ or ‘Takanari’ (**Supplementary Fig. S10**). Genes involved in protein phosphorylation and defense response were significantly enriched in the 1,139 genes (GO: 0006468, adjusted *P*-value = 1.67E-06 and GO: 0006952, adjusted *P*-value = 2.92E-05, **Supplementary Table S5**). For example, a *cis*-eQTL could explain the expression dynamics polymorphism of *Os01g0537250* that could be specifically observed in young plants (**Fig. 3E, F**). The expression depended on time of day in both parental models, but scaled age was only important in the ‘Koshihikari’ model (**Fig. 3E, F** and **Supplementary Fig. S9**). Together, the eQTL affected 393 genes depending on the time of day and 275 genes depending on scaled age (**Supplementary Fig. S10**), suggesting that our method of eQTL detection clarifies the genetic basis of varietal differences in environmental responses. The genetic regions around the SSR markers RM3701 and RM5824 on chromosome 11 influence many time-of-day- and scaled-age-dependent genes (**Supplementary Tables S6** and **S7**). GO-enrichment analysis revealed that these genes significantly enriched disease resistance genes (**Supplementary Table S8**).

**Fig. 4:**
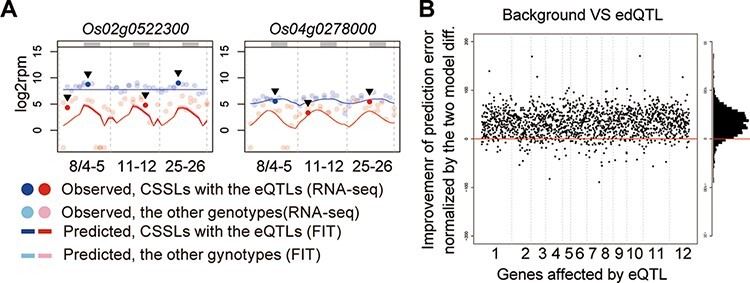
eQTL-based prediction of gene expression dynamics under different environments. (A) Examples of prediction of expression dynamics in Kizugawa in 2016 based on environmental information and eQTLs in transplant set 1. Points in intense colors emphasized by arrowheads indicate samples harboring eQTLs different from their BGs and light colors indicate samples harboring eQTLs identical to their BGs. The upper gray bars indicate dark periods (global solar radiation < 0.3 kJ m^−2^ min^−1^). (B) Effects of eQTLs on gene expression prediction dynamics in Kizugawa in 2016. Genes influenced by eQTLs are in the order of the positions on the chromosomes along the horizontal axis.

In the eQTL evaluation step, we first compared the prediction performances based on the eQTL model and background genotype model (BG model) under different environments from where training data were obtained for model development. ‘Koshihikari’, ‘Takanari’ and the CSSLs of two transplant sets were cultivated in a field different from that used in 2015 and sampled in August 2016; 139 RNA-Seq datasets were obtained (**Supplementary Fig. S2C** and **Table S2**). Environmental factors differed between the 2015 and 2016 fields (**Supplementary Fig. S11A**). For 91.8% of the 1,675 genes affected by the eQTL, the prediction was improved by the eQTL model compared with the BG model (**Fig. 4A, B** and **Supplementary Fig. S11B**). Furthermore, the prediction of the expressed genes for the validation dataset showed comparable accuracy to that of the training dataset (**Supplementary Fig. S12**). Thus, the identified eQTLs could explain the expression dynamics polymorphisms in different years and locations.

**Fig. 5:**
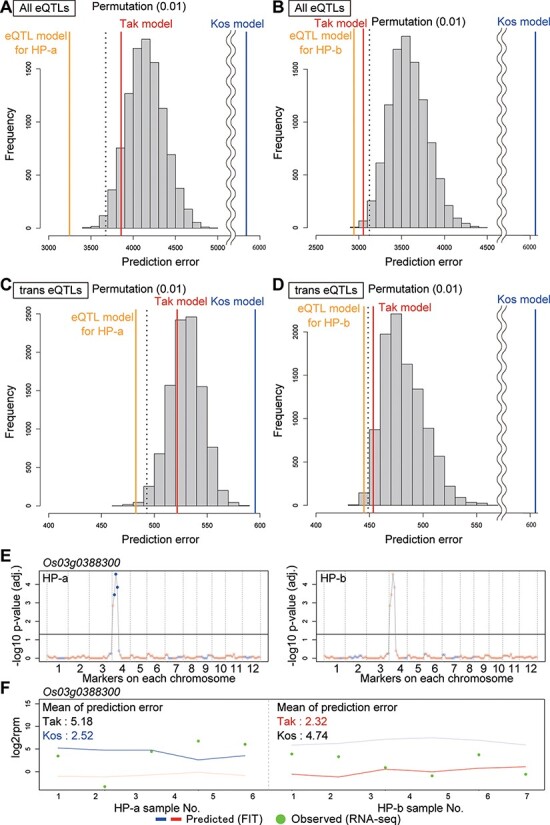
eQTL-based prediction in cultivars with more complex genotypes than the CSSLs. (A–D) Prediction accuracy of gene expression regulated by all eQTLs (A, B) and *trans*-eQTLs (C, D) based on eQTL model for HP-a (A, C) and HP-b (B, D). The blue, red and orange vertical lines indicate the sums of prediction errors based on ‘Koshihikari’, ‘Takanari’ and eQTL models. The histogram shows the distribution of the sums of prediction errors based on the eQTL model in 10,000 permutations of markers in HP-a or HP-b genomes. The dashed vertical line indicates the 0.1% percentile of the distribution. (E) eQTL for *Os03g0388300* and genotypes of HP-a and HP-b. Dark blue points indicate significant ‘Koshihikari’-type markers. (F) Prediction of *Os03g0388300* expression in HP-a and HP-b. Intense color lines are applied models for HP-a and HP-b.

For further verification, two BILs between ‘Koshihikari’ and ‘Takanari’ (HP-a and HP-b) (Adachi et al. 2013, 2019) were cultivated at Takatsuki, Japan, in 2015, and five and six RNA-Seq datasets were obtained for HP-a and HP-b, respectively (**Supplementary Fig S2B** and **Table S2**), and the lines carried ‘Koshihikari’ alleles with the ‘Takanari’ genetic background in 16.3% and 19.9% of their markers, respectively (**Supplementary Fig S2A**). To evaluate the performance of the eQTL-based prediction, we calculated the sum of prediction errors of all eQTL-influenced genes using eQTL, ‘Koshihikari’, and ‘Takanari’ models. Overall, the eQTL model provided the optimal prediction (**Fig. 5A****–****D**). Permutation analysis of markers in HP-a and HP-b genomes revealed the significant benefits of the eQTL models even for *trans*-eQTLs (*P* < 0.01) (**Fig. 5A****–****D**). For instance, because the genotype of the *trans*-eQTL for *Os03g0388300* comprised the ‘Koshihikari’ allele in the genome of HP-a and the ‘Takanari’ allele in the genome of HP-b (**Fig. 5E**), ‘Koshihikari’ and ‘Takanari’ models were used to predict *Os03g0388300* expression in the eQTL models for HP-a and HP-b, respectively. The expression of *Os03g0388300* fluctuated with time and it was higher in ‘Koshihikari’ than in ‘Takanari’ (**Supplementary Fig. S13A**). The prediction of *Os03g0388300* expression based on the eQTL models was better than the prediction based on the BG models (**Fig. 3F**). Regarding *OsKS3* (*Os04g0611700*) (Sakamoto 2004) in HP-b, the prediction based on the eQTL model was worse than that based on the BG model (**Supplementary Fig. S13B, C**). True eQTLs might therefore exist around SSR markers 52 and 53, which were not substituted with the ‘Koshihikari’ allele in HP-b (**Supplementary Fig. S13B**). Such challenges may be observed in some eQTL-based predictions for the BILs, because several eQTLs identified by the CSSLs can be unlinked to the substituted genome regions in the BILs. Finally, we concluded that our approach successfully identified loci linked to expression polymorphisms in field conditions.

## Discussion

Our eQTL approach scans a broader range of conditions (**Fig. 1A**) but it is less sensitive when focusing on a specific condition. This is because eQTLs are expected to show the same effect on gene expressions in all samples used in the conventional method. Conversely, in our approach some eQTLs were expected to affect gene expressions in specific samples, depending on their environments. The lower sensitivity of the our eQTL detection relative to that of the conventional eQTL approach might explain the smaller fraction of *trans*-eQTL compared with that of *trans*-eQTL in previous studies (13.3% and 62–71% (Wang et al. 2010, 2014, Kuroha et al. 2017), respectively) because *trans*-eQTLs have generally smaller effect sizes than *cis*-eQTL/eQTLs (Wang et al. 2010, 2014).

Phenotypic plasticity plays key roles in plant’s environmental adaptation (Sultan 1995, Fournier-Level et al. 2016). Nevertheless, as this is difficult to study in natural environments, the genetic architecture of phenotypic plasticity under field conditions remains largely unveiled. The eQTLs identified by our novel method improve the understanding of the genetic architecture underlying the expression dynamics polymorphisms between ‘Koshihikari’ and ‘Takanari’ in the field. Polymorphism(s) between Koshihikari and Takanari around RM3701 and RM5824 influenced the expression of many time-of-day- and scaled-age-dependent genes, including genes related to disease resistance (**Supplementary Tables S7** and **S8**). Defense responses in plants can vary, depending on the circadian rhythm and age (Lu et al. 2017). These eQTLs might contribute to the time-of-day- and age-dependent differences in the innate immunity, between Koshihikari and Takanari.

In addition, our method showed that rice gene expression dynamics can be predicted based on genotypic and meteorological data. In crop breeding, polymorphisms of environmental responses result in unexpected performance of a bred crop under environments differing from experimental fields. Because the transcriptome can be beneficial for trait prediction (Skelly et al. 2009, Horinouchi et al. 2017, Kaur et al. 2017, Kremling et al. 2018) and our approach allows transcriptome predictions under various conditions, it contributes to crop breeding and for understanding plant systems.

In summary, we developed a novel approach of eQTL identification to reveal the genomic architecture causing varietal differences in expression dynamics under field conditions, which is difficult to assess by conventional eQTL methods. By applying the method to field-grown rice (‘Koshihikari’, ‘Takanari’ and their CSSLs), we succeeded in the identification of 1,675 eQTLs leading to polymorphisms in expression dynamics under field conditions. The fidelity was verified by predicting gene expression under environments different from the training environments and in rice cultivars with more complex genotypes than the CSSLs. Our method will contribute to an understanding of the genomic basis of varietal differences in environmental responses.

## Materials and Methods

### Overview of eQTL identification and verification

Our approach consisted of four steps (**Fig. 1B**). First, the parent rice lines and their descendants were cultivated in a paddy field and sparsely sampled at several time points for RNA-Seq (Time-series RNA-Seq, **Fig. 1B** and **Supplementary Fig. S1**). Second, by using the RNA-Seq data, scaled age, meteorological information and ‘FIT’ (Iwayama et al. 2017), parental prediction models were developed to describe environmental responses in ‘Koshihikari’ and ‘Takanari’ in terms of gene expression (Prediction model development, **Fig. 1B**). Most gene expression dynamics would be identical among the CSSLs and the parent with the same BG. Thus, we used ‘Koshihikari’- and ‘Takanari’-background CSSLs as well as their parents to develop the parental models (**Fig. 1B** and **Supplementary Fig. S1**). Third, the dependency of expression dynamics polymorphisms on genetic variation was statistically evaluated based on comparisons between predictive gene expression and observed gene expression in CSSLs assuming that genetic variation of SSR markers leads to expression dynamics polymorphisms (**Fig. 2B** and **Supplementary Fig. S7**) (eQTL detection, **Fig. 1B**). Fourth, by integrating the parental models and eQTL information, gene expression dynamics was predicted based on environmental and genotypic information (Evaluation of eQTLs). The detail of eQTL identification is described in Supplementary information.

### Plant materials

We cultivated the following rice (*O. sativa*) lines: the *japonica* variety ‘Koshihikari’, the *indica* variety ‘Takanari’, 78 reciprocal CSSLs (40 ‘Koshihikari’-background lines, except for SL1213, and 38 ‘Takanari’-background lines, except for SL1306) (Takai et al. 2014) and two ‘Takanari’-background BILs (HP-a and HP-b) (Adachi et al. 2013, 2019). Initially, we tried to use all CSSL lines. However, genotype validation using RNA-Seq revealed that the genotypes of the lines initially labeled as SL1213 and SL1306 were nearly identical to that of SL1210 and SL1308, respectively. Therefore, we treated the plants as SL1213 and SL1306; the two CSSLs, SL1213 and SL1306, could not be included in this study. Each variety and line were sown in nursery trays. Approximately 1 month after sowing, seedlings were transplanted to a paddy field at Takatsuki, Japan (34°51′19″N, 135°37′51″E) in 2015 and at Kizugawa, Japan (34°44′03″N, 135°50′18″E) in 2016. To consider the effect of plant age in the prediction of gene expression dynamics, four and two transplant sets were prepared in 2015 and 2016, respectively (**Fig. 1C** and **Supplementary Fig. S2B, C**). Seed sowing and transplanting were conducted according to the following schedules. In 2015, transplant set 1: Seed sowing date ‘April 3^rd^, 2015’, Transplanting date ‘May 1^st^, 2015’; transplant set 2: Seed sowing date ‘April 17^th^, 2015’, Transplanting date ‘May 8^th^, 2015’; transplant set 3: Seed sowing date ‘May 1^st^, 2015’, Transplanting date ‘May 22^nd^, 2015’; transplant set 4: Seed sowing date ‘May 15^th^, 2015’, Transplanting date ‘June 5^th^, 2015’. In 2016, transplant set 1: Seed sowing date ‘April 21^st^, 2016’, Transplanting date ‘May 16^th^, 2016’; transplant set 2: Seed sowing date ‘May 19^th^, 2016’, Transplanting date ‘June 8^th^, 2016’.

### Sampling and RNA extraction

Sixteen sets (2015) and three sets (2016) of bihourly sampling for 22 h, from 16:00 on one day to 14:00 on the next, were conducted on the following dates (**Supplementary Fig. S2B, C** and **Table S2**): 5–6 May, 16–17 June, 22–23 June, 29–30 June, 6–7 July, 13–14 July, 20–21 July, 27–28 July, 3–4 August, 19–20 August, 24–25 August, 31 August to 1 September, 7–8 September, 14–15 September, 21–22 September and 28–29 September in 2015; and 4–5, 11–12 and 25–26 August in 2016. We applied a stratified randomization strategy to the sampling schedule to avoid biased sampling of each line across seasons. We separated individual plants into four and two groups in each transplant set in 2015 and 2016, respectively, each containing a similar number of individuals per line. Then, the order of sampling was randomized in each group. Sampling was begun 2 weeks after transplantation in 2015 and in August in 2016 (22 May, 25 May, 6 July and 20 July in 2015 and 4 August in 2016). According to the sampling schedule, two plants from the 82 genotypes of each transplant set were sampled. Due to the withering of aged rice, several samples were missed in the late periods of the cultivation (Gray points in **Supplementary Fig. S2B**). Transplant set and sampling time for each sample are listed in **Supplementary Table S2**. The youngest fully expanded leaf from each plant was collected, immediately frozen in liquid nitrogen and stored at −80°C until RNA isolation for RNA-Seq. Individual plants were only sampled once to avoid wounding response. Therefore, all the RNA-Seq data were obtained from independent plants. Frozen samples were homogenized with TissueLyser II (Qiagen, Hilden, Germany), and total RNA was then extracted using the Maxwell 16 LEV Plant RNA Kit (Promega, Madison, WI, USA) and Maxwell 16 Automated Purification System (Promega). }The concentration of RNA was measured using the Quant-iT RNA Assay Kit, broad range (Thermo Fisher Scientific, Waltham, MA, USA).

### RNA-Seq library preparation and sequencing

RNA-Seq libraries were prepared as described previously (Kashima et al. 2020) except that mRNA enrichment was performed with enzymatical degradation of abundant RNAs such as rRNAs (Nagano et al. 2015). The detail of RNA-Seq library preparation is described in Supplementary information. Sequencing of 50-bp single ends using HiSeq 2500 (Illumina, San Diego, CA, USA) was carried out by Macrogen (Seoul, South Korea).

### Calculation, normalization and quality control of RNA-Seq count data

Quality control and mapping of RNA-Seq data were conducted as described in Ishikawa et al. (2017), with reference sequences of IRGSP-1.0_transcript (Kawahara et al. 2013). The detail of the quality control and mapping of RNA-Seq data are described in Supplementary information.

Of the 926 samples from 2015 and 143 samples from 2016, we used 887 and 139 RNA-Seq transcriptome datasets, respectively, with more than 10^5^ total read counts for all genes, except for the targets of selective depletion (**Supplementary Fig. S3A**). We then filtered out rarely detected genes (number of samples with read count > 0; ≤ 20% of all samples in the 2015 dataset) from the following analyses to focus on the 23,924 expressed genes only (**Supplementary Fig. S3B**).

### Correlation plot of transcriptomes

Pearson correlation coefficients of transcriptome data for all pairwise comparisons of the 854 samples from 2015 were calculated as follows:

(1) \begin{equation*}{\rho _{m,n}} = {{\mathop \sum \nolimits_i \left( {{y_{i,m}} - {{\bar y}_m}} \right)\left( {{y_{i,n}} - {{\bar y}_n}} \right)} \over {\sqrt {\mathop \sum \nolimits_i {{\left( {{y_{i,m}} - {{\bar y}_m}} \right)}^2}} \sqrt {\mathop \sum \nolimits_i {{\left( {{y_{i,n}} - {{\bar y}_n}} \right)}^2}} }}\end{equation*}

where *ρ_m,__n_* denotes the Pearson’s correlation coefficient between samples *m* and *n*. Mean log2-transformed rpms are denoted as ${\bar y_m}$ and ${\bar y_n}$, respectively.

The heatmap representing the correlations in the time-series order was drawn with the ‘image.plot’ function in the R package ‘fields’ (version 9.0) (Nychka et al. 2017).

### Meteorological data

Meteorological data were obtained from weather stations close to the fields in Takatsuki and Kizugawa. Data of average air temperature per 10 min at the Hirakata Weather Station (34°48′53″N, 135°39′04″E, 4.87 km away from the Takatsuki field) in 2015 for Takatsuki and at Nara Weather Station (34°40′27″N, 135°49′56″E, 6.77 km away from the Kizugawa field) in 2016 for Kizugawa were obtained from the Japan Meteorological Agency. Data of air temperature per minute for the FIT (version 0.0.4) (Iwayama et al. 2017) library were prepared by linear interpolation of the data per 10 min with the ‘approxfun’ function in R (version 3.4.2) (R Core Team 2017). Data of global solar radiation per minute at the Osaka Weather Station (34°40′55″N, 135°31′05″E, 21.84 km away from the Takatsuki field) in 2015 for Takatsuki and at the Nara Weather Station (34°40′27″N, 135°49′56″E, 6.77 km away from the Kizugawa field) in 2016 for Kizugawa were also obtained from the Japan Meteorological Agency. The data at the study fields and the data from the close meteorological stations were significantly correlated (*P* < 0.001); Pearson correlation coefficients of daily mean temperature and radiation in August in 2015 were 0.99 and 0.97 (*n* = 31), respectively, and Pearson correlation coefficients of hourly mean temperature and radiation in August in 2016 were 0.97 and 0.99 (*n* = 744 for 31 days), respectively.

### GO-enrichment analysis for hour- and scaled-age-dependent genes

The database of GO terms in Rice Annotation Project Database (Sakai et al. 2018) was used. GO-enrichment analysis was conducted with a function: fisher.test. Then, the adjustment for multiple comparisons against *P*-values was performed using the Benjamini–Hochberg method [54] using the p.adjust function in R. Finally, we listed GO terms with the smallest adjusted *P*-value (<0.05) (**Supplementary Tables S4** and **S5**).

## Supplementary Material

pcab088_Supp

## Data Availability

All datasets generated and/or used in this study are available from PRJDB7234. The scripts used in this study are available from https://github.com/naganolab/Rice_edQTL-analysis_and_edQTL-based-prediction.
