# Supplementary material for: Genomic Basis of Transcriptome Dynamics in Rice under Field Conditions
Source: Plant Cell Physiol. 2021 Jun 16;62(9):1436–45. doi: 10.1093/pcp/pcab088 (PMC8600290; doi:10.1093/pcp/pcab088)
Supplement: pcab088_Supp [file pcab088_supp.zip › pcp-2021-e-00065-File008.pdf]

Supplementary Information for

Genomic basis of transcriptome dynamics in rice under field conditions

Makoto Kashima, Ryota L. Sakamoto, Hiroki Saito, Satoshi Ohkubo, Ayumi Tezuka, Ayumi Deguchi, Yoichi Hashida, Yuko Kurita, Koji Iwayama, Shunsuke Adachi and Atsushi J. Nagano

Correspondence to: anagano@agr.ryukoku.ac.jp

Materials and Methods

RNA-Seq library preparation

An automated liquid handling system Freedom EVO 150 (TECAN, Zurich, Switzerland) and a thermal cycler ODTC 384 (INHECO, Martinsried, Germany) were utilized to prepare the RNA-Seq library of 384 samples simultaneously. The protocol of RNA-Seq library preparation is described below. We enzymatically degraded abundant RNAs such as rRNAs in the leaves, which cause wasteful consumption of sequence reads (Supplementary Fig. 14). Aliquots of 252 and 192 types of 100-μM 60-mer antisense DNAs (IDT, San Jose, CA, USA) covering rRNAs and the top eight most frequently-detected transcripts (*Osp1g00110.1*, *Osp1g00180.1*, *Osp1g00420.1*, *Osp1g00170.1*, *Osp1g00340.1*, *Osp1g00330.1*, *Osp1g00600.1*, and *Os11t0707000-02*) in the RNA-Seq dataset of *O. sativa* leaf were mixed in equal amounts (SDRNA oligo pool) (Morlan et al., 2012; Nagano et al., 2015). One microgram of total RNA, 1.5 μL of the SDRNA oligo pool, 3 μL of 5× hybridization buffer (0.5 M Tris-HCl (pH 7.4) and 1 M NaCl), and RNase-free water were mixed to a total volume of 10 μL. The mixture was incubated to anneal the antisense DNAs with the target transcripts using the thermal cycler ODTC 384 with the following program: 95 °C for 2 min, slow cooling to 45 °C (0.1 °C/s), and 45 °C for 5 min. Subsequently, 1 μL of Tli Thermostable RNase H (TaKaRa, Kusatsu, Japan), 2 μL of 10× RNase H digestion buffer [500 mM Tris-HCl (pH 7.4), 1 M NaCl, and 200 mM MgCl], and 7 μL of RNase-free water were added and mixed well. This mixture was incubated at 45 °C for 30 min to selectively digest the RNA of the DNA/RNA hybrid double strand. Then, 24 μL of AMPure XP (Beckman Coulter, Brea, CA, USA) beads was added and the mixture was purified in a 384-well magnetic plate following the manufacturer's instructions. The RNA was then eluted with 14 μL of RNase-free water.

Ten microliters of the RNA solution, 1 μL of DNaseI (5 unit/μL), 1 μL of 10× DNaseI buffer (both Promega), 1 μL of 100 mM DTT (Invitrogen, Carlsbad, CA, USA), and 7 μL of RNase-free water were mixed well. This mixture was incubated at 37 °C for 30 min to degrade the SDRNA oligo pool. Subsequently, 24 μL of AMPure XP beads was added and purified according to the manufacturer's instructions. The RNA was eluted with 14 μL of RNase-free water. Five microliters of the purified mRNA obtained was mixed with 4 μL of 5× SS buffer (Invitrogen), and 1 μL of 100 mM DTT. Fragmentation of mRNA was carried out at 94 °C for 4.5 min and immediately cooled on ice. Subsequently, 0.6 μL of 100 μM random primer $(N)_6$ (Promega) and 0.9 μL of RNase-free water were added to the mixture, which was incubated at 50 °C for 5 min and immediately chilled on ice to relax the secondary structure of the RNA. The fragmented RNA with random hexamers and the reverse transcription master mix [1 μL of 100 mM DTT, 0.4 μL of dNTPs (25 mM each; Promega), 0.1 μL of SuperScript IV (Invitrogen), 0.2 μL of actinomycin D (1000 ng/μL) (Nacalai Tesque, Kyoto, Japan), and 5.9 μL of RNase-free water] were mixed. For the reverse transcription step, the mixture was incubated at 25 °C for 10 min, followed by 50 min at 50 °C. SuperScript IV was inactivated by heating the mixture at 75 °C for 15 min. Subsequently, 24 μL of AMPure XP and 12 μL of 99.5% ethanol were added, and the mixture was purified according to the manufacturer's protocol. The reverse transcription product was eluted with 14 μL of RNase-free water. The purified DNA/RNA hybrid solution without beads and the second-strand synthesis master mix [2 μL of 10× Blue Buffer (Enzymatics, Beverly, MA, USA), 1 μL of dUTP/NTP mix (Fermentas, Burlington, Canada), 0.5 μL of 100 mM DTT, 0.5 μL of RNase H (Enzymatics), 1 μL of DNA polymerase I (Enzymatics), and 5 μL of RNase-free water] were mixed. This mixture was incubated at 16 °C for 4 h, and then purified with 24 μL of AMPure XP

according the manufacturer's protocol. The purified dsDNA was eluted with 14 μL of RNase-free water. Five microliters of the dsDNA solution was used in the following step. End-repair, A-tailing, and adapter ligation were carried out using the KAPA Hyper prep kit (KAPA BIOSYSTEMS, Wilmington, MA, USA) with 1/10× volume of the solutions according the manufacturer's protocol. One microliter of 0.1 μM Y-shape adapter (Nagano et al., 2015) was used in the adapter ligation step for 15 min. Then, size selection of the ligation product was performed with 5.5 μL of AMPure XP. The purified dsDNA was eluted with 10 μL of RNase-free water and a second round of size selection was performed with 10 μL of AMPure XP. The size-selected ligation product was eluted with 15 μL of 10 mM Tris-HCl (pH 8.0). One microliter of uracil DNA glycosylase (UDG; Enzymatics) was added to the size-selected ligation product. The mixture was incubated at 37 °C for 30 min to exclude the second-strand DNA. For library amplification, 2 μL of the UDG-digested DNA, 1 μL of 2.5 μM index primer (Nagano et al., 2015), 1 μL of 10 μM universal primer (Nagano et al., 2015), 0.5 μL of RNase-free water, and 5 μL of KAPA HiFi HotStart ReadyMix (2×) (KAPA BIOSYSTEMS) were mixed. The DNA fragments with the adapters and an index sequence were amplified using a thermal cycler with the following program: denaturation at 94 °C for 2 min, 16 cycles at 98 °C for 10 s, 65 °C for 30 s, and 72 °C for 30 s for amplification, and 72 °C for 5 min for the final extension. Two rounds of size selection were then performed to remove adapter dimers with equal volume of AMPure XP to the library solution. The purified library was eluted with 10 μL of RNase-free water. The concentration of each library was measured by qPCR using the KAPA SYBR FAST qPCR Master Mix (2×) Kit (KAPA BIOSYSTEMS) and the LightCycler 480 II (Roche Diagnostics, Basel, Switzerland) to mix equal amounts of libraries. One microliter of the mixed library was used for electrophoresis with the

Bioanalyzer 2100 and the Agilent High Sensitivity DNA kit (Agilent Technologies, Santa Clara, CA, USA) to check for quality.

Calculation, normalization and quality control of RNA-Seq count data. In order to trim low-quality bases and adapter sequences from reads, all obtained reads were processed using Trimmomatic (version 0.3.3) (Bolger et al., 2014) using the following parameters: TOPHRED33 ILLUMINACLIP:TruSeq3-SE.fa:2:30:10 LEADING:19 TRAILING:19 SLIDINGWINDOW:30:20 AVGQUAL:20 MINLEN:40. This procedure removed adapter sequences (ILLUMINACLIP:TruSeq3-PE.fa:2:30:10), as well as leading and trailing low quality (Q < 19) or N bases (LEADING:19 TRAILING:19). It also trimmed the reads when the average quality per base dropped below 20 with a 30-base wide sliding window (SLIDINGWINDOW:30:20). These trimmed reads were then mapped to the reference sequences of IRGSP-1.0_transcript (Kawahara et al., 2013; Sakai et al., 2018), rRNAs, and transcripts coded in the mitochondria and chloroplast genomes (NC_001320.1, NC_011033.1) using RSEM (version 1.3.0) (Li and Dewey, 2011) and Bowtie (version 1.1.2) (Langmead et al., 2009) with default parameters. Expected counts of each gene in the RSEM outputs were used in R (version 3.4.2) (R Core Team, 2017) for the following analysis. According to a previous study (Kamitani et al., 2016), we removed the effects of misassigned reads in multiplex sequencing experiments with HiSeq 2500 as:

$$c_{i,m,n} = \begin{cases} \tilde{c}_{i,m,n} - 0.0005 \times C_{i,n} \left(\tilde{c}_{i,m,n} > 0.0005 \times C_{i,n}\right), \\ 0 \left(\tilde{c}_{i,m,n} \leq 0.0005 \times C_{i,n}\right), \end{cases} \quad (1)$$

where $c_{i,m,n}$ and $\tilde{c}_{i,m,n}$ denote corrected and raw read counts of gene i in sample m in RNA-Seq library pool n, respectively. $C_{i,n}$ denotes the sum of read counts of gene i in RNA-Seq library pool n.

Then, read counts of gene i in sample m in different libraries were merged,

followed by read count normalization based on total read counts of transcripts except for the antisense-oligo targets (reads per million: rpm):

$$r_{i,m} = \frac{c_{i,m}}{C_m} \times 10^6, \tag{2}$$

$$y_{i,m} = \log_2\left(r_{i,m} + 0.1\right), \tag{3}$$

where, $c_{i,m}$, $r_{i,m}$, and $y_{i,m}$ denote merged corrected read counts, normalized read counts, and the log2-transformed value of read counts of gene i in sample m, respectively. C_m denotes total read counts of transcripts except for the antisense-oligo targets in sample m.

Identification of specific 'Koshihikari' and 'Takanari' single nucleotide polymorphisms. The paired-end reads of 'Koshihikari' (SRR1630927) and 'Takanari' (DRR018360) genomes were trimmed with Trimmomatic (version 0.33) (Bolger et al., 2014) using the parameters stated in the previous section, except for TruSeq3-PE.fa for adapter trimming. The reads were then mapped to the reference genome (IRGSP-1.0, plastid: NC_001320.1, mitochondrion: NC_011033.1) (Kawahara et al., 2013) using Bowtie2 (version 2.2.9) with option -N1, which allows a mismatch in seed alignment (Langmead and Salzberg, 2012). By using SAMtools (version 1.3.1) (Li et al., 2009), the reads mapped at multiple loci and the duplicated reads, except for each representative read, were discarded. Picard (version 2.1.0) (https://broadinstitute.github.io/picard/) was used to add read group information to each SAM file. All SAM files of each sample were merged and a VCF file was generated using SAMtools (version 1.3.1) (Li et al., 2009) and BCFtools (version 1.1) (Li et al., 2009). Subsequently, single nucleotide polymorphism (SNP) calling was conducted using VCFtools (version 0.1.14) (Danecek et al., 2011) with option --minDP5, which indicates that all alleles supported by more than four reads are reported, in order to obtain reliable SNP calls. The VCF file generated was analyzed using R (version 3.4.2) (R Core Team, 2017) and the R package "vcfR" (version

1.5.0) (Knaus and Grünwald, 2017). We selected 15,117 homozygous SNPs distinguishing ‘Koshihikari’ and ‘Takanari’ genotypes for the following analysis of genotype confirmation.

Genotype confirmation. In previous studies, the genotypes of the CSSLs and BILs were examined based on the genotypes provided by 141 SSR markers (Takai et al., 2014). A SNP call based on our RNA-Seq was insufficient for accurate detection of genome-wide genetic variations, due to low sequencing depth (Supplementary Fig. 4). Thus, to confirm the genotype of each sample, we estimated each SSR marker genotype based on the SNPs obtained by RNA-Seq but did not use the SNPs themselves. First, to type ‘Koshihikari’- or ‘Takanari’-type SNPs in each RNA-Seq sample, the trimmed RNA-Seq reads of each sample were mapped to the reference genome as described in the previous section using Bowtie2 (version 2.2.9). Afterward, SNP calling was conducted using VCFtools (version 0.1.14) (Danecek et al., 2011) with option --minDP3, which indicates that all alleles supported by more than two reads are reported, in order to obtain reliable SNP calls. To estimate each SSR marker genotype in our samples, ‘Koshihikari’- and ‘Takanari’-type SNPs were counted from the SSR marker position until one of the counts reached “7” (Supplementary Fig. 4A, B). In the cases where the SNPs around an SSR marker position were too sparse, all the SNPs in ±1.5 Mbp from each SSR marker were counted (Supplementary Fig. 4A, C). Then, each estimated SSR marker was categorized into ‘Koshihikari’-type, ‘Takanari’-type, or undetermined based on most of its SNP counts (Supplementary Fig. 4A-C). Any samples of the estimated genotypes not perfectly matching the SL1201, SL1229, or SL1321 genotypes might be due to the low density of SNPs around the substituted SSR markers or to doubtful SNP calls. For the samples with estimated genotypes similar to one of these lines, imputation of estimated SSR marker

genotypes was conducted (Supplementary Fig. 4D). Finally, each sample was labeled with the genotype of the most similar SSR marker composition. Thus, 865 samples were confirmed as ‘Koshihikari’, ‘Takanari’, or CSSLs and 11 samples as BILs in 2015, and 139 samples as ‘Koshihikari’, ‘Takanari’, or CSSLs in 2016 (Supplementary Fig. 3D).

Conversion of age to scaled age. Logarithmic expressions of more than a thousand genes followed a linear function of age-in-days in our previous work (Nagano et al., 2012). To keep our model as simple as possible, we also employed the simplest function for the age-in-days dependent change in the present study. To consider the differences based on plant age at heading among genotypes, we defined scaled age as below. Scaled age correlated with gene expression more than unscaled age-in-days (Supplementary Fig. 6), suggesting that using scaled age in our predictive model development is better. Under the assumption that rice ages linearly before heading at various rates among genotypes, flowers at scaled age 85, and age at the same rate after flowering, the scaled age of sample *m* was defined as:

$$a_m = \begin{cases} 85\dfrac{d_m - d_m^S}{d_m^F - d_m^S} & (d_m \leq d_m^F), \\ d_m - d_m^F + 85 \; (d_m > d_m^F), \end{cases} \tag{5}$$

where d_m is the sampling date, d_m^S is the seeding date, and d_m^F is the flowering date of each line with genotype and seeding date identical to that of sample *m.*

Calculation of precision weights for each observed normalized read count (log2rpm). Precision weights were utilized in the training step of FIT (version 0.0.4) (Iwayama et al., 2017) to deal with over-dispersion of RNA-Seq data (Iwayama et al., 2017). Precision weight for each observed log2rpm value was calculated based on the residuals from the smoothed time-series as in the voom method (Iwayama et al., 2017; Law et al., 2014) (Supplementary Fig. 15). First, for each gene, spline interpolation against time and

observed log2rpm was conducted with the "splinefun" function in R, to infer the function value $s_{iG}(t)$ for interpolation of observed log2rpm of gene i at time t in $G \in \{\mathrm{K}, \mathrm{T}\}$, where K and T denote 'Koshihikari' and 'Takanari'-background genotypes, respectively (Supplementary Fig. 15A). Then, residual standard deviation of gene i in G-background genotype $\sigma_{i,G}$ was calculated as:

$$\sigma_{i,G} = \sqrt{\frac{1}{|M_G|} \sum_{m \in M_G} \left(s_{i,G}(t_m) - y_{i,m}\right)^2}, \tag{6}$$

where t_m denotes sampling time of sample m. M_G and $|M_G|$ denote indices of samples whose background genotypes are G and its size, respectively.

The mean log2-transformed read count of gene i in G-background genotype was then calculated as:

$$\bar{c}_{i,G} = \frac{1}{|M_G|} \sum_{\mathrm{m} \in \mathrm{M_G}} y_{i,m} + \frac{1}{|M|} \sum_{m \in M} \log_2 C_m + 1 - \log_2 10^6, \tag{7}$$

where M denotes sample indices in both background genotypes.

A LOWESS curve (Cleveland, 1981) was then fitted to the square-root of residual standard deviations as a function of mean log2-transformed read counts, resulting in $l()$, which represents the trend between log2-transformed read counts and square-root of residual standard deviations (Supplementary Fig. 15B).

Similar to $\bar{c}_{i,G}$, smoothed log2rpm of gene i in sample m was converted to smoothed log2-transformed read counts of gene i in sample m as:

$$\hat{c}_{i,m} = s_{i,G_m}(t_m) + \log_2 C_m + 1 - \log_2 10^6, \tag{8}$$

where G_m denotes the background genotype of sample m.

Finally, precision weight for observed log2rpm of gene i in sample m was calculated as (Supplementary Fig. 15C):

$$w_{i,m} = \frac{1}{l(\hat{c}_{i,m})^4}. \quad (9)$$

Prediction model development for 'Koshihikari' and 'Takanari'. A prediction model for gene expression dynamics in 'Koshihikari' and 'Takanari' was developed with the R package "FIT" (version 0.0.4) (Iwayama et al., 2017). In this section, we describe how "FIT" developed a prediction model.

There are several strategies for the integrative analysis of the transcriptome and meteorological data to investigate environmental responses in field conditions (Cronn et al., 2017; Izawa et al., 2011; Richards et al., 2012; Sjödin et al., 2008; Wilkins et al., 2016). In the FIT package, we employed the statistical modeling approach for a comprehensive relationship among these data and for understanding the effects of age and circadian clock of plants. Therefore, we used the transcriptome data in combination with the meteorological data, namely air temperature and global solar radiation, which were acquired from close meteorological stations. Because there was no remarkable disease or pest damage during cultivation and no difference in fertilization among samples, the model did not include these effects.

The log2rpm of the normalized expression for gene *i* of sample *m* was described by a simple linear model:

$$y_{i,m} = \alpha_i + \boldsymbol{x}_{i,m}^T \boldsymbol{\beta}_i + \epsilon_{i,m}, \quad (10)$$

where α_i, $\boldsymbol{x}_{i,m}$, and $\boldsymbol{\beta}_i$ are a constant term, a vector of eight explanatory variables, and regression coefficients, respectively. The third term $\varepsilon_{i,m}$ is the independently and identically distributed noise drawn from a Gaussian distribution. The explanatory variables were plant's scaled age, circadian clock, optional variable, response to environmental stimuli, and the interactions between scaled age and circadian clock and between scaled age and response to environmental stimuli. The optional variable was not

used in this study. The plant's scaled age corresponded to the scaled number of days after transplanting. See section "Conversion of age to scaled age" for the definition of scaled age. The values of the scaled age were normalized to mean 0 and variance 1. The circadian clock, with the peak at an arbitrary time, was represented by two variables corresponding to its cosine and sine components:

$$\begin{aligned} &\cos\left(\frac{2\pi t_m}{24}\right), \\ &\sin\left(\frac{2\pi t_m}{24}\right), \end{aligned} \tag{11}$$

where t_m is the time at which sample m was obtained. Similarly, the interaction between scaled age and circadian clock of a plant was represented by two variables.

Although we employed a linear model for simplicity, the actual gene expression can exhibit more complex responses. The complexity of fluctuations in gene expression is mainly explained by the nonlinearity of the response to the environmental stimuli, which is the cumulative sum of the nonlinearly transformed environmental stimuli for a given period. Such cumulative sum of temperature, for example, is designated as cumulative temperature and it is widely used for the prediction of reproduction timing and vegetation in ecology and of harvest timing in agriculture (Kira, 1948; Schwartz, 2014). By applying this to gene expression, the environmental response of gene i in sample m can be described as:

$$\hat{e}_{i,m} = \sum_{\tau=t_m-p_i}^{t_m} g_i(\tau) \max\left(0, \tanh\left(\pm \exp\left(\gamma_i^{(f)}\right)\left(e_\tau - \theta_i^{(f)}\right)\right)\right) \times \sqrt{\exp\left(-2\gamma_i^{(f)}\right) + 1}, \tag{12}$$

where p_i is the period during which gene expression was affected by an environmental stimulus, e_τ is the value of a meteorological parameter at time τ normalized to mean 0 and variance 1, $\theta_i^{(f)}$ is the response threshold to the stimulus, and $\gamma_i^{(f)}$ is the parameter that

controls the shape of the response (Supplementary Fig. 16). Here, the meteorological data consisted of air temperature and global solar radiation. Of these parameters, the one yielding the highest likelihood was selected for each gene in the optimization procedure. In the limit $\gamma_i^{(f)} \rightarrow \infty$, a gene responds to an environmental stimulus in a dose-independent manner, that is, the response is constant if a meteorological parameter exceeds the threshold (Supplementary Fig. 16). Conversely, as this parameter approaches minus infinity, the response also approaches the dose-dependent manner (Supplementary Fig. 16). The sign of the value in the hyperbolic function determines whether the gene responds to an environmental stimulus larger than (positive) or smaller than (negative) the threshold. This sign was decided in the optimization procedure based on the parameter likelihood. The last term $\sqrt{\exp\left(-2\gamma_i^{(f)}\right) + 1}$ ensures that the scale of the response is largely independent of this parameter. The function $g_i(\tau)$ is a gate function that explains the time-of-day-specific environmental responses. A gate function is defined as:

$$g_i(\tau) = n_i\left(\tanh\left(\exp\left(\gamma_i^{(g)}\right)\left(\cos\left(2\pi(\tau - \psi_i)\right)/24 - \theta_i^{(g)}\right)\right)\right), \qquad (13)$$

where $\gamma_i^{(g)}$controls the shape of the gate as $\gamma_i^{(f)}$ in the environmental response, $\theta_i^{(g)}$ determines the opening length of the gate, and $n_i()$ is the function which normalizes an input value $[0,1]$. This gate function approaches a sinusoidal and rectangular wave with the small and large values of $\gamma_i^{(g)}$, respectively. If the value of $\theta_i^{(g)}$ is smaller than -1, the gate is a constant value in the limit $\gamma_i^{(g)} \rightarrow \infty$, that is, the gate is always open (Supplementary Fig. 16).

It can be considered that some explanatory variables might contribute to neither the explanation nor the prediction of the expression of many genes. Thus, we simultaneously preformed the optimization and variable selection of the regression coefficients by using

group lasso (Yuan et al., 2006). Let I be the index set for scaled age, environmental response, and the interaction between scaled age and environmental response. The cost function to be minimized is thus defined as:

$$L(\boldsymbol{\beta}) = \sum_{m \in M} \left(\hat{y}_{i,m} - y_{i,m}\right)^2 + \lambda \left(\sum_{k \in I} \zeta_k |\beta_k| + \zeta_c \sqrt{\beta_{cos}^2 + \beta_{sin}^2} + \zeta_{dc} \sqrt{\beta_{dcos}^2 + \beta_{dsin}^2} \right), (14)$$

where λ and $\boldsymbol{\zeta}$ are the regularization parameter and the adaptive weights, respectively. The values of the adaptive weights are decided as in the adaptive lasso (Zou, 2006) or the adaptive group lasso(Wang and Leng, 2008). Further, the adaptive weights were used to absorb differences in the degrees of freedom by multiplying the covariates of the environmental response and interaction between the environmental response and scaled age by seven, as seven free parameters were considered. We selected the value of λ as the largest value for which the cross validation (CV) error was smaller than the sum of the minimum CV error and its standard error.

The values of the parameters related to environmental responses, which are $p_i, \theta_i^{(f)}, \gamma_i^{(f)}$, and the sign of the value in the hyperbolic function in Eq. (12), and $\theta_i^{(g)}, \gamma_i^{(g)}$, and ψ_i in Eq. (13), are selected by the Nelder-Mead algorithm (Nelder and Mead, 1965). Because the likelihood function is complex and has multiple local maxima, a grid search was performed before optimization. In each step of the optimization by the Nelder-Mead algorithm, we performed an ordinary least square regression instead of group lasso (Eq. (14)) to reduce the computational cost.

'Koshihikari' and 'Takanari'-prediction models were developed based on time-series RNA-Seq data of the 'Koshihikari'-background CSSLs and 'Koshihikari' and of

the ‘Takanari’-background CSSLs and ‘Takanari’ in 2015, respectively (Supplementary Fig. 2B). Values of the expressed genes (a matrix of log2rpm for each sample), sample attributes (a matrix of sampling year, month, day, hour, and min and scaled age for each sample), weather data (a matrix of air temperature and global solar radiation at every minute in 2015), and precision weights for the matrix of log2rpm of each sample (Supplementary Fig. 16) were prepared. Details on the parameters for ‘FIT’ are listed in https://github.com/naganolab/Rice_eQTL-analysis_and_eQTL-based-prediction/analysis.R. As a result, the functions $f_{i,G}()$ were yielded for gene i. A log2rpm of gene i of sample m was predicted as (Supplementary Fig. 16):

$$\hat{y}_m = f_{i,G_m}(t_m, a_m, \boldsymbol{e}), \tag{15}$$

where $\boldsymbol{e}$ denotes meteorological data (air temperature and global solar radiation) at every minute.

Detection of genes with expression dynamics polymorphism. To increase detection power, we focused on genes with expression dynamics polymorphism. Euclidean distances of gene i between ‘Koshihikari’- and ‘Takanari’-prediction models (d_i) were calculated as:

$$d_i = \sqrt{\sum_{m \in M} \left(f_{i,\mathrm{K}}(t_m, a_m, \boldsymbol{e}) - f_{i,\mathrm{T}}(t_m, a_m, \boldsymbol{e})\right)^2}. \tag{16}$$

As a result, 3696 genes with $d_i > 40$ were defined as genes with expression dynamics polymorphism (Supplementary Fig. 3E).

Detection of eQTLs. According to background genotypes, the sum of residual errors between the observed log2rpm and predicted log2rpm of gene i in the ‘Koshihikari’- or ‘Takanari’-models (E_i) were calculated as (Supplementary Fig. 3A):

$$E_{i,m} = \left|\hat{y}_{i,m} - y_{i,m}\right|, \tag{17}$$

$$E_i = \sum_{m \in M} E_{i,m}\,, \tag{18}$$

where E_{im} denotes the residual error between the observed log2rpm and predicted log2rpm of gene i in sample m on the assumption that the 'Koshihikari'- or 'Takanari'-type gene expression dynamics shown by sample m is determined based on background genotype G_m. Similarly, on the assumption that the eQTL for gene i exists around SSR marker g, the sum of residual errors between the observed log2rpm and predicted log2rpm of gene i in the 'Koshihikari'- or 'Takanari'-models $\tilde{E}_{i,g}$ were calculated as follows (Supplementary Fig. 3A):

$$\tilde{E}_{i,m,g} = \left| f_{i,Q_{g,m}}(t_m, a_m, \boldsymbol{e}) - y_{i,m} \right|, \tag{19}$$

$$\tilde{E}_{i,g} = \sum_{m \in M} \tilde{E}_{i,m,g}\,, \tag{20}$$

where $\tilde{E}_{i,m,g}$ denotes the residual error between the observed log2rpm and predicted log2rpm of gene i in sample m and $Q_{g,m}$ denotes genotype of SSR marker g in sample m. Thus, $f_{i,Q_{gm}}(t_m, a_m, \boldsymbol{e})$ denotes predicted log2rpm in sample m assuming the existence of eQTL for gene i around SSR marker g. The improvement in residual errors assuming that eQTL for gene i exists around g was therefore calculated as follows (Supplementary Fig. 3A, B):

$$I_{i,g} = E_{i,m} - \tilde{E}_{i,m,g}. \tag{21}$$

To evaluate the statistical significance of the improvement in the sum of residual errors, 1000 permutations of samples with 'Koshihikari'- and 'Takanari' genetic background were performed (Supplementary Fig. 7D). For each permutation, the improvement in residual errors on the assumption that eQTL for gene i exist around SSR marker g was calculated (Supplementary Fig. 2B). Then, we generated the null distribution of improvements in residual errors by fitting log-normal distribution to

obtained improvements using the fitdist function in the R package “fitdistrplus” (Delignette-Muller and Dutang, 2015), with the fitting method of quantile matching of 70% and 1% (Supplementary Fig. 7B). For almost all genes with expression dynamics polymorphism, the 99.9 percentiles of the fitted log-normal distributions were higher than (2863/3696 genes) or almost the same as those of the distribution obtained by the permutations (Supplementary Fig. 7C), indicating that the *p*-value obtained from log-normal distribution was conservative. Thus, the *p*-value for the improvement in the sum of residual errors for gene *i* on the assumption that an eQTL affecting gene expression dynamics of gene *i* exists around SSR marker *g* was obtained based on the null distribution.

The adjustment for multiple comparisons against *p*-values was performed with the Benjamini-Hochberg method (Benjamini and Hochberg, 1995) using the p.adjust function in R. Finally, we defined the markers with the smallest adjusted *p*-value (< 0.05) in each peak for each gene as the eQTL affecting each gene expression dynamic.

The eQTL-based prediction of gene expression dynamics (eQTL model). To predict gene expression with eQTL information, the ‘Koshihikari’- or ‘Takanari’-model was selected based on the genotype of the eQTL for eQTL-affected genes and of genetic backgrounds for the other expressed genes. Let $\mathcal{G}_\mathrm{i}$ denote the set of eQTLs affecting gene *i*. We selected the model for prediction of log2rpm of gene *i* in sample *m* as:

$$g_{i,m} = \begin{cases} \tilde{G}_m, & \left(\exists g \in \mathcal{G}_i, Q_{g,m} \neq G_m\right), \\ G_m, & \text{otherwise}, \end{cases} \tag{22}$$

where $\widetilde{\mathrm{G}}_m$ denotes the opposite genotype to that of the genetic background in sample *m*, that is, $\tilde{G}_m = \mathrm{K}$, if sample *m* is ‘Takanari’-background, and vice versa. Then, predicted log2rpm of gene *i* in sample *m* was calculated as:

$$\hat{y}_{i,m}^{\mathrm{ED}} = f_{i,g_{i,m}}(t_m, a_m, \boldsymbol{e}). \tag{23}$$

Statistical test for the accuracy of gene expression dynamics prediction in BILs. The sums of prediction errors of all genes affected by each eQTL for the BILs based on the 'Koshihikari' model (E_{K}), 'Takanari' model (E_{T}), and eQTL model (E^{ED}) were calculated as:

$$E_G = \sum_{i \in I^{\mathrm{ED}}, m \in M^{\mathrm{B}}} \left| f_{i,G}(t_m, a_m, \boldsymbol{e}) - y_{i,m} \right|, \tag{24}$$

$$E^{\mathrm{ED}} = \sum_{i \in I^{\mathrm{ED}}, m \in M^{\mathrm{B}}} \left| \hat{y}_{i,m}^{\mathrm{ED}} - y_{i,m} \right|, \tag{25}$$

where M^{B} denotes the indices of BIL samples and I^{ED} denotes the genes affected by the eQTL.

To evaluate the statistical significance of improvement in the sums of prediction errors, 10,000 permutations were performed for the markers in each BIL genome. In each permutation, the sums of prediction errors of all genes affected by the eQTLs for the BILs were calculated with the eQTL model. Then, the value corresponding to percentile 0.1 of the sums of prediction errors was calculated.

Reference

Benjamini, Y., and Hochberg, Y. (1995) Controlling the False Discovery Rate : A Practical and Powerful Approach to Multiple Testing. *J R Stat Soc Ser B*. 57: 289–300.

Bolger, A.M., Lohse, M., and Usadel, B. (2014) Trimmomatic: a flexible trimmer for Illumina sequence data. *Bioinformatics*. 30: 2114–2120.

Cleveland, W.S. (1981) LOWESS: A Program for Smoothing Scatterplots by Robust Locally Weighted Regression. *Am Stat*. 35: 54.

Cronn, R., Dolan, P.C., Jogdeo, S., Wegrzyn, J.L., Neale, D.B., St. Clair, J.B., et al. (2017) Transcription through the eye of a needle: Daily and annual cyclic gene expression variation in Douglas-fir needles. *BMC Genomics*. 18.

Danecek, P., Auton, A., Abecasis, G., Albers, C.A., Banks, E., DePristo, M.A., et al. (2011) The variant call format and VCFtools. *Bioinformatics*. 27: 2156–2158.

Delignette-Muller, M.L., and Dutang, C. (2015) fitdistrplus : An R Package for Fitting Distributions. *J Stat Softw*. 64: 1–34.

Iwayama, K., Aisaka, Y., Kutsuna, N., and Nagano, A.J. (2017) FIT: Statistical modeling tool for transcriptome dynamics under fluctuating field conditions. *Bioinformatics*. 33: 1672–1680.

Izawa, T., Mihara, M., Suzuki, Y., Gupta, M., Itoh, H., Nagano, A.J., et al. (2011) Os-*GIGANTEA* Confers Robust Diurnal Rhythms on the Global Transcriptome of Rice in the Field. *Plant Cell*. 23: 1741–1755.

Kamitani, M., Nagano, A.J., Honjo, M.N., and Kudoh, H. (2016) RNA-Seq reveals virus-virus and virus-plant interactions in nature. *FEMS Microbiol Ecol*. 92: 1–11.

Kawahara, Y., de la Bastide, M., Hamilton, J.P., Kanamori, H., McCombie, W.R., Ouyang, S., et al. (2013) Improvement of the Oryza sativa Nipponbare reference genome using next generation sequence and optical map data. *Rice*. 6: 4.

Kira, T. (1948) On the altitudinal arrangement of climatic zones in Japan—a contribution to the rational land utilization in cool highlands. *Kanti-Nogaku*. 2: 143–173.

Knaus, B.J., and Grünwald, N.J. (2017) vcfr: a package to manipulate and visualize variant call format data in R. *Mol Ecol Resour*. 17: 44–53.

Langmead, B., and Salzberg, S.L. (2012) Fast gapped-read alignment with Bowtie 2. *Nat Methods*. 9: 357–359.

Langmead, B., Trapnell, C., Pop, M., and Salzberg, S.L. (2009) Ultrafast and memory-efficient alignment of short DNA sequences to the human genome. *Genome Biol*. 10: R25.

Law, C.W., Chen, Y., Shi, W., and Smyth, G.K. (2014) voom: precision weights unlock linear model analysis tools for RNA-seq read counts. *Genome Biol*. 15: R29.

Li, B., and Dewey, C.N. (2011) RSEM: accurate transcript quantification from RNA-Seq data with or without a reference genome. *BMC Bioinformatics*. 12: 323.

Li, H., Handsaker, B., Wysoker, A., Fennell, T., Ruan, J., Homer, N., et al. (2009) The sequence alignment / map (sam) Format and SAMtools. *Bioinformatics*. 25: 2078–2079.

Morlan, J.D., Qu, K., and Sinicropi, D. V. (2012) Selective depletion of rRNA enables whole transcriptome profiling of archival fixed tissue. *PLoS One*. 7: e42882.

Nagano, A.J., Honjo, M.N., Mihara, M., Sato, M., and Kudoh, H. (2015) Detection of plant viruses in natural environments by using RNA-Seq. *Plant Virol Protoc Methods Mol Biol*. 1236: 89–98.

Nagano, A.J., Sato, Y., Mihara, M., Antonio, B. a., Motoyama, R., Itoh, H., et al. (2012) Deciphering and prediction of transcriptome dynamics under fluctuating field conditions. *Cell*. 151: 1358–1369.

Nelder, J.A., and Mead, R. (1965) A Simplex Method for Function Minimization. *Comput J*. 7: 308–313.

R Core Team (2017) R: A language and environment for statistical computing. R Foundation for Statis-tical Computing, Vienna, Austria. [WWW Document].

Richards, C.L., Rosas, U., Banta, J., Bhambhra, N., and Purugganan, M.D. (2012) Genome-wide patterns of Arabidopsis gene expression in nature. *PLoS Genet*. 8.

Sakai, H., Lee, S.S., Tanaka, T., Numa, H., Kim, J., Kawahara, Y., et al. (2018) Rice annotation project database (RAP-DB): an integrative and interactive database for rice genomics. *Plant Cell Physiol*. 54: 1–11.

Schwartz, M. (2014) Phenology : an integrative environmental science. Springer.

Sjödin, A., Wissel, K., Bylesjö, M., Trygg, J., and Jansson, S. (2008) Global expression profiling in leaves of free-growing aspen. *BMC Plant Biol*. 8.

Takai, T., Ikka, T., Kondo, K., Nonoue, Y., Ono, N., Arai-Sanoh, Y., et al. (2014) Genetic mechanisms underlying yield potential in the rice high-yielding cultivar Takanari, based on reciprocal chromosome segment substitution lines. *BMC Plant Biol*. 295: 14.

Wang, H., and Leng, C. (2008) A note on adaptive group lasso. *Comput Stat Data Anal*. 52: 5277–5286.

Wilkins, O., Hafemeister, C., Plessis, A., Holloway-Phillips, M.M., Pham, G.M., Nicotra, A.B., et al. (2016) EGRINs (Environmental gene regulatory influence networks) in rice that function in the response to water deficit, high temperature, and agricultural environments. *Plant Cell*. 28: 2365–2384.

Yuan, M., Yuan, M., and Lin, Y. (2006) Model selection and estimation in regression with grouped variables. *J R Stat Soc Ser B*. 68: 49--67.

Zou, H. (2006) The adaptive lasso and its oracle properties. *J Am Stat Assoc*. 101: 1418–1429.
